# Attacking the Intruder at the Gate: Prospects of Mucosal Anti SARS-CoV-2 Vaccines

**DOI:** 10.3390/pathogens11020117

**Published:** 2022-01-19

**Authors:** Kacper Karczmarzyk, Małgorzata Kęsik-Brodacka

**Affiliations:** 1 Department of Bacterial Genetics, Institute of Microbiology, Faculty of Biology, University of Warsaw, 02-096 Warsaw, Poland; 2 National Medicines Institute, 00-725 Warsaw, Poland; m.kesik@nil.gov.pl

**Keywords:** COVID-19, SARS-CoV-2, mucosal vaccines

## Abstract

The sudden outbreak of the severe acute respiratory syndrome coronavirus-2 (SARS-CoV-2) pandemic in December 2019 caused crises and health emergencies worldwide. The rapid spread of the virus created an urgent need for the development of an effective vaccine and mass immunization to achieve herd immunity. Efforts of scientific teams at universities and pharmaceutical companies around the world allowed for the development of various types of preparations and made it possible to start the vaccination process. However, it appears that the developed vaccines are not effective enough and do not guarantee long-lasting immunity, especially for new variants of SARS-CoV-2. Considering this problem, it is promising to focus on developing a Coronavirus Disease 2019 (COVID-19) mucosal vaccine. Such a preparation applied directly to the mucous membranes of the upper respiratory tract might provide an immune barrier at the primary point of virus entry into the human body while inducing systemic immunity. A number of such preparations against SARS-CoV-2 are already in various phases of preclinical and clinical trials, and several of them are very close to being accepted for general use, constituting a milestone toward pandemic containment.

## 1. Introduction

Coronavirus Disease 2019 (COVID-19), caused by severe acute respiratory syndrome coronavirus-2 (SARS-CoV-2), was first reported at the end of 2019 in Wuhan, China. Its rapid spread across the globe has resulted in the worst pandemic since the Spanish Flu (1918) and caused the world to experience a huge health crisis. At the time this review was published (17 January 2022), there were approximately 329 million confirmed cases and over 5.5 million recorded deaths due to COVID-19 [1]. These numbers produce a constant need to seek effective strategies to protect against the disease. In 2020, we saw the start of the race to develop an effective and safe vaccine by research teams at universities and pharmaceutical companies. Currently, there are over 300 anti-SARS-CoV-2 vaccine candidates under different stages of preclinical and clinical development [2], and 10 preparations are approved for use by the World Health Organization (WHO) [3]. During the post-approval period, research has shown that although those vaccines protect against severe disease, they do not provide long-term protection or limit the spread of the virus and are not fully effective against emerging new variants of SARS-CoV-2 [4,5,6]. Indeed, the highly transmissible delta variant causes asymptomatic infection and occasionally illnesses in vaccinated people, most likely due to increased growth potential and waning immunity. Hence, the effectiveness of approved vaccines still needs to be improved, and 139 candidates are in different phases of clinical trials, according to the WHO [2]. The approved vaccines and great majority of preparations under development are designed to be administered via the intramuscular route (IM) to provide high production of systemic antibodies to trigger against systemic viral infection [7]. This route of administration, despite being the most common method of immunization, is not optimal in view of protection against pathogens entering our body via mucosal routes.

Apart from the fact that vaccine development is an expensive process, administration of intramuscular preparation is associated with additional costs, including logistics (need for cold-chain transport), the device needed for injection and the need for trained medical personnel, which significantly reduces the chances of fast mass immunization, especially in underdeveloped countries [8]. Parenteral vaccines are also known to cause injection fear, not only among children but among adults as well, which, unfortunately, can discourage part of a community from undergoing vaccination. Many people experience fear and pain from needle procedures. This is often a cause of fainting and a lot of stress and can lead to the avoidance of healthcare in the future [9]. As mentioned earlier, IM vaccines mainly induce a systemic immune response, and since we know that SARS-CoV-2 infects humans through mucosal surfaces of the respiratory tract, it should be considered whether this type of reaction is sufficient [10]. Taking into consideration that SARS-CoV-2 infects the body through the mucous membranes of the upper respiratory tract, it would be logical to expect that effective vaccines against SARS-CoV-2 would be delivered through mucosal routes and function by mimicking natural infection.

## 2. Mucosal Vaccine as a Promising Approach in the Fight against the Pandemic

Pathogens infecting the respiratory tract are one of the leading causes of global mortality [11]. The ongoing pandemic only reminds us of the enormous threat posed by respiratory mucosa infections, especially because there is no universal vaccine to protect against them. In the current situation, we need to focus on the development of new, more effective vaccines, as much is needed to prevent infection by pathogens such as SARS-CoV-2 [12]. Control of infectious diseases is possible due to vaccines, which have a huge impact on combatting pathogens and stopping them from spreading in the community. Effective immunization is achieved when an adequate level of protection to reduce transmission of the pathogen is obtained [13]. The mucosa, which is a barrier between the organism and the external environment, is the direct point of entry into the body of the majority of infectious pathogens [14]. Humans are infected by this route by pathogens such as *Streptococcus pneumoniae*, *Haemophilus influenzae* B, respiratory syncytial virus (RSV), influenza virus, *Helicobacter pylori*, and, among others, the entire coronavirus family [12]. All of these factors are the cause of high and still increasing morbidity and mortality, despite the wide availability of vaccines against most of them, and may indirectly be because the majority of available immunization preparations are delivered subcutaneously or intramuscularly, which allows for the generation of systemic immune protection but is not sufficient to induce a local immune response to the antigen presented on the mucosal tissue surface [15]. For this reason, there is growing interest in mucosal vaccine development, which in general can have a significant impact on the control of pathogens infecting via mucous membranes. The surfaces of mucous membranes, for example, the gastrointestinal tract and intranasal or pulmonary spaces, constitute the largest area of exposure in the human body [16]. They are also much thinner and more permeable than the skin, which makes them an ideal site for pathogens to enter the body. The mucosal immune system, which protects against penetration of intruding agents, acts as prevention. Its main line of defense is secretory IgA (sIgA), which has the ability to effectively protect against systemic infection. Nonetheless, most vaccines are still administered intramuscularly or subcutaneously, which, in general, protects the host only when systemic invasion occurs and does not allow for a rapid immune response during antigen presentation at the point of entry. Contrary to the aforementioned disadvantages and problems of parenteral vaccines, immunization via mucous membranes (e.g., by the oral or nasal route) has a number of significant advantages, while having fewer limitations at the same time [17]. The most important ones are listed in Table 1.

Despite having many theoretical advantages, there are only nine mucosal vaccines approved for use in humans. These are mainly oral preparations (8 out of 9) designed to protect against *Vibrio cholerae* (Dukoral, Shanchol, Vaxchora), influenza A and B viruses (FluMist), *Salmonella typhimurium* (Typhi Vivotif), poliovirus (Biopolio, mOPV/tOPV) and rotavirus (Rotateq, Rotarix). All of these preparations have shown that mucosal immunization allows induction of a strong immune response, including mucosal sIgA and serum IgG, as well as stimulation of memory T cells. The findings demonstrate that it is a very feasible strategy and is certainly worthy of further analysis [18]. Contrary to the rapid development and production of injectable vaccines based on new technologies, such as those containing protein subunits combined with specific adjuvants and those containing the pathogen’s DNA and RNA, all of the mucosal vaccines mentioned above are whole-cell inactivated or live attenuated vaccine formulations. The lack of progress in this matter is in part due to the constant search for a suitable platform for the administration of such preparations, the high probability of degradation of subunit antigens (especially when administered orally) and the negligible amount of proven and safe mucosal adjuvants [12,19,20].

## 3. Nanotechnology-Based Mucosal Vaccine Platforms Overview

As mentioned above, mucosal vaccines approved to date are based on live attenuated and whole cell inactivated pathogens. They are not formulated with the use of specific adjuvants, as this role is played by pathogen-associated molecular patterns (PAMPs), and bacterial cells and viruses act as the delivery systems themselves. The lack of a new, optimal form of a vaccine, such as subunit vaccines (used in traditional vaccines) consisting of only defined components of the pathogen (e.g., structural proteins or enzymes), is one of the reasons for the poor technological progress of this type of vaccine. Induction of an immune response by subunit vaccines is a very interesting approach; however, it requires combining the antigen with an appropriate adjuvant and selecting the correct delivery system depending on the chosen mucosal area. The mucosal environment generates many difficulties that prevent proper stimulation of immune cells. In the case of oral preparations, the delivered antigen should reach the intestines intact, where it would be presented on mucous membranes. This requires tolerance to the low pH of the stomach, as well as the sudden increase in alkalinity in the duodenum. The antigen must also be well protected against proteolytic digestive enzymes because of the route. Preparations administered intranasally, despite the lack of such drastic fluctuations in pH as in the digestive system, also face obstacles in the form of large volumes of mucosal secretions, in which the antigen concentration is reduced; mucociliary cleansing and, most importantly, poor capture efficiency by antigen presenting cells (APC) are also issues [17,21].

A promising prospect of overcoming these obstacles may be the use of nanotechnology-based delivery systems, which are currently widely analyzed in research laboratories. Nanoscale carriers are already being used in approved vaccines and in those analyzed in clinical trials [22]. The currently used or further investigated nanocarriers include liposomes, virus-like particles (VLPs), nanogels, immunostimulatory complexes (ISCOMs) and inclusion bodies. In addition to carrying a specific antigen, these carriers are capable of modulating immune responses through a variety of interactions with APCs. The ideal carrier, in addition to the antigen, carries a specific adjuvant that allows enhancement of the immune response to the presented antigen and allows them to be delivered together to the right location at the right time [23]. In addition, some nanocarriers, such as VLPs, act as adjuvants themselves [24].

### 3.1. Liposomes and Immunostimulatory Complexes

Liposomes are carriers composed of one or more phospholipid membranes surrounding an aqueous core. Due to the structural properties of liposomes, they allow free design of vaccine formulations based on them. Therefore, it is possible to produce liposomes with a specific size, lamellarity and surface charge to induce the desired immune response. Moreover, the main building block of liposomes (phospholipids) is a component of mammalian cell membranes, making them nontoxic and completely biodegradable [25]. To date, most research associated with attempts to develop an effective liposome-based delivery system has focused on parenteral vaccines. Nevertheless, their potential for use in mucosal vaccines has also been widely analyzed [26]. The possibilities of using this nanocarrier have already been explored in the context of combatting pathogens such as influenza virus, human immunodeficiency virus, hepatitis B virus, *Vibrio cholerae*, *Pseudomonas aeruginosa* and *Yersinia pestis* [27]. Liposomes are known to allow the transfer of peptides, proteins and DNA, helping to induce cell-mediated immunity by interacting with epithelial cells. The surface charge of liposomes is the main factor determining their adjuvant character, and compared to neutral and negatively charged liposomes, positively charged (cationic) liposomes interact most strongly with epithelial cells [28,29,30,31]. A cationic surface charge enables mucoadhesion, which allows for stronger interaction with the mucosa of the gastrointestinal tract and better adherence to cells, which in general enhances internalization [32]. However, it is worth mentioning that cationic liposomes show greater toxicity than anionic liposomes and can also cause damage and inflammation. Liposomes are also susceptible to damage caused by lipases and bile salts, which can lead to the premature release of antigens [33,34]. Therefore, to maximize their potential in the context of nanocarriers, their stability and durability, especially in the gastrointestinal tract, should be improved. Considering that the use of liposomes to date has had positive effects, such as generating a cellular and systemic immune response to influenza virus haemagglutinin [35], it is worthwhile to analyze and improve them further.

Quite similar carriers to liposomes are immunostimulatory complexes (ISCOMs). ISCOMs comprise phospholipids, cholesterol, saponins and an antigen to form cage-like micellar molecules [36]. The ability to entrap antigens through apolar interactions renders ISCOMs promising immunostimulants. These nanoparticles have considerable potential for use as an antigen delivery system, bearing in mind that thus far, they have been used together with antigens from influenza virus, herpes simplex virus and Newcastle disease virus [37,38,39]. Difficulties with antigen incorporation into nanocarriers have led many studies to include ISCOMs as an adjuvant component of the vaccine. The obtained results were usually similar to those with encapsulation of the antigen inside the carrier [40]. Although the production of pure ISCOMs for use as adjuvants is much simpler, this approach lacks the benefits of antigen encapsulation, which is very important for mucosal delivery systems. Therefore, unmodified ISCOMs are only used for intranasal and parenteral vaccines with antigens only or together with other adjuvants [41,42,43].

### 3.2. Virus-like Particles

Virus-like particles are structures composed of specific structural viral proteins without viral genetic material [44]. Complex VLPs mimic the form of a complete virus and, more importantly, can mimic an authentic viral infection. They have been extensively analyzed in the context of their use in oral formulations against viruses and tumors. These studies have shown that VLP-based vaccines are effective in inducing cellular and humoral immune responses both in the mucosa and throughout the body [45,46,47]. VLPs are also relatively simple to obtain under laboratory conditions using recombinant viral proteins and can be expressed in many expression systems, such as bacteria, yeasts and plants [48,49,50]. The expression of VLPs in plants allows for their purification at low cost, and freeze-dried plant tissues containing VLPs can be directly administered to animals, inducing effective immune responses, which offers prospects for creating inexpensive edible vaccines for humans [51,52].

### 3.3. Nanogels

Nanogels are produced with the use of natural or synthetic polymers by cross-linking hydrophilic polymer networks. The properties of the polymers from which they are synthesized confer nanogels with a number of advantages in terms of their use as a delivery system, the most important of which are that their size can be freely modified, that they generate a large surface area with numerous exposed functional groups, and that they ensure high stability and biocompatibility, as well as high load capacity [53,54]. It has also been shown that due to their physicochemical nature, nanoparticles are immunologically active, target specific cells and effectively protect antigens against degradation [55]. Regarding the possibility of use in mucosal vaccines, the best analyzed nanogel is cholesteryl group-bearing pullulan (CHP), which binds perfectly to epithelial cells and is effectively absorbed. It has also been shown that despite a lack of adjuvant activity, CHP is capable of inducing a local and systemic immune response. These features indicate that CHP may be considered a universal antigen delivery system in intranasal vaccines. In addition, the described nanocarrier is safe, and research shows that the antigens it carries do not remain in the olfactory bulb and do not accumulate in the brain, excluding the risk of neurotoxicity. All of the above information suggests that CHP is a good candidate for intranasal vaccine preparation that should be assessed in further clinical trials [56,57,58].

### 3.4. Inclusion Bodies

In addition to the mentioned vectors, the use of inclusion bodies (IBs) as an antigen delivery system seems to be an interesting approach. IBs are insoluble aggregates of misfolded peptide chains that may accumulate while recombinant proteins are overexpressed by genetically modified bacteria [59]. Several advantages of the use of genetically modified bacteria (e.g., they show rapid and controlled growth, are able to efficiently express foreign genes and are relatively effortless to manipulate) make them a widely used protein production system [60]. The use of IBs as an antigen delivery system has been proposed due to their unique properties [61]. The particulate structure of IBs makes them well suited for mucosal vaccination. This form of antigen favors oral immunization, as it is per se protected from digestion in the gastrointestinal tract.

The advantage of using IBs is that only a few steps are required to obtain a carrier rich in the selected vaccine antigen. IBs are also able to induce protection and both systemic and mucosal immune responses without adjuvant coadministration. Mice orally administered IBs containing the classical swine fever virus E2 antigen twice in the absence of adjuvant developed both systemic and mucosalimmune response [62]. The simplicity of expression of foreign antigens in bacterial expression systems, combined with the ease of isolating IBs enriched with them, render this method an attractive antigen delivery system for oral immunization and would provide effective generation of an immune response [63,64].

## 4. Live Vector Systems

### 4.1. Lactic Acid Bacteria

Lactic acid bacteria (LAB) are gram-positive, non-pathogenic microorganisms that are studied in the context of, inter alia, the development of new and safe recombinant protein (including vaccine antigens) delivery systems. These bacteria represent an alternative to the whole range of mostly pathogenic attenuated microorganisms used as carriers. Recombinant LAB strains are able to induce a strong, both systemic and mucosal, immune response against the carried antigens. Intense research on *Lactococcus lactis* and species of the *Lactobacillus* genus confirms the potential use of these bacteria as vaccine adjuvants, immunostimulants and drug delivery systems [65,66]. LAB are able to survive in the digestive tract, thanks to which they reach the intestines undamaged, and then colonize them without causing negative effects on the host organism. This ability makes them an attractive option for an oral antigen delivery system [67].

LABs are already being analyzed for their use in immunization against SARS-CoV-2. Keikha et al. designed vaccine preparations based on self-amplifying RNA lipid nanoparticles (saRNA LNPs), saRNA-transfected *Lactobacillus plantarum* LNPs and saRNA-transfected *Lactobacillus plantarum*. It has been shown that all variants can express the SARS-CoV-2 virus S-protein at both mRNA and protein levels. Oral immunization of mice with these vaccines resulted in the secretion of antibodies capable of neutralizing the alpha and delta variants of SARS-CoV-2 [68].

### 4.2. Plants

The production of vaccine antigens derived from plants can become cost-effective system for the large-scale production of human therapeutic proteins [60,69]. The use of plants to produce vaccine antigens ensures that all post-translational modifications are completed in the protein of interest since plants possess the expression, folding, assembly and glycosylation machinery required to achieve the antigen’s structure and biological activity. Plant-based vaccines can effectively stimulate humoral and cellular responses at mucosal and systemic sites. The use of plants for vaccine antigen production eliminates its potential contamination with animal pathogens, as plant cell cultures are not susceptible to mammalian viral pathogens and, conversely, plant viruses do not infect human cells [70]. There are some plant-based vaccines for COVID-19 in the pipeline. One of the plant-derived vaccines for COVID-19 developed by the Medicago company [71] is currently after the third phase of clinical trials [2] and initiated the regulatory filling process. The overall vaccine efficacy rate against all variants of SARS-CoV-2 is 71%.

Plants could be used as bioreactors for production and as delivery systems for vaccine antigens. In this approach, orally immunogenic recombinant proteins expressed in an edible plant may be orally administered without processing, including costly purification steps [72]. A key feature of plant-made vaccines is that the vaccine antigens are bioencapsulated by plant cell walls, which protects them from degradation in the stomach’s acidic environment.

## 5. Adjuvants–Enhancement of the Local Immune Response

Contrary to parenteral immunization, induction of an immune response on the mucosa usually requires the administration of a higher dose of antigen. It happens due to the dilution of the vaccine preparation in the mucus and its partial excretion by ciliary movements and mucus in the airways [73]. Passing through the mucus layer and reaching the surface of the mucosal tissue is necessary for inducing local production of IgA antibodies. In the case of preparations administered orally, the low pH environment, as well as nucleases and proteases present in various sections of the gastrointestinal tract, also prevent the antigen from reaching the immune sites. In many cases, these physical and biochemical obstacles lead to the ineffective induction of an immune response on mucosal tissue. To overcome them and to more effectively engage mucosal immune cells, oral and nasal vaccines are often supplemented with appropriate adjuvants [74]. Adjuvants are components of vaccine preparations that are meant to either enhance or modulate the humoral or cellular immune response to the presented antigen [75]. Using an effective adjuvant in conjunction with an antigen can provide great benefits, including preventing the body’s tolerogenic responses to the antigen, recruitment and activation of APCs and the engagement of a wide range of other cells actively involved in the processes of the immune response [12]. Immune cell populations abundantly present in mucous tissue, such as innate lymphoid cells, mucosal-associated invariant T cells or natural killer T cells, play a very important role in building an immune response and can be effectively activated by adjuvants [76,77,78,79,80,81,82,83]. Unfortunately, only a few effective and safe adjuvants have been known so far. However, those targeting cells, such as microfold (M) cells and dendritic cells (*Escherichia coli* double-mutant heat-labile toxin), or activating invariant natural killer T cells (α-galactosylceramide) offer very promising prospects [12].

Immunization of the lungs with liquid and dry vaccines induced an immune response both systemically and on the mucosa in preclinical studies. However, the low titers of mucosal IgA antibodies emphasized the weakness of this response. Therefore, the use of adjuvants is necessary to obtain effective immunization by the mucosal route [84,85]. Preparing mucosal vaccines by combining selected antigens with appropriate adjuvants seems to be an easy process. In addition, it is known that the use of an adjuvant reduces the required dose of antigen and significantly enhances the immune response evoked against it [86]. As the vast majority of approved adjuvants have been studied in the context of their use in conventional vaccines, it is not fully understood how they act in mucosal immune responses. The best known mucosal adjuvants are cholera toxin (CT) and *Escherichia coli* heat-labile enterotoxin (LT) [87], the adjuvant effect of which is based on interaction with the surface of dendritic cells, enhancing induction of B cell clones [88]. Due to the high affinity for the mucosa, a number of polymers are used as adjuvanted carriers, such as chitosan [89]. Additionally, the liposomes mentioned in the section about carriers are widely analyzed in this context [90]. ISCOMs also seem to be effective adjuvants for mucosal vaccines [91]. Another approach may be the use of specialized molecules, e.g., lectins, that facilitate targeting of the antigen to surface markers of epithelial and dendritic cells, which increases the efficiency of APC antigen uptake [92].

Aluminum salts are another commonly known and used adjuvant. Human vaccines against pathogens such as human papilloma virus, hepatitis A and B viruses, influenza type B, tetanus or diphtheria contain alum [93]. This component has already been tested in potential preparations against COVID-19. Gao et al. used adjuvant aluminum hydroxide in a vaccine based on an inactivated SARS-CoV-2 virus. This preparation generated a strong humoral response in rhesus macaques, which gave them complete protection against the virus, and no lung immunopathology was found after its use [94]. The protein subunit (S protein or receptor binding domain) vaccine with aluminum, developed by Liang et al., generated high levels of serum IgG and provided long-term action of B cells in mice [95]. Despite their safety and efficacy in eliciting a humoral immune response, aluminum compounds used as adjuvants have drawbacks. The main limitations are poor immunostimulation of cellular immune responses and limitations in use in preparations intended to protect against intracellular pathogens. Alum is also not effective in supporting the mechanisms aimed at activating and colonizing T and B lymphocytes in mucosal tissues [96].

Hence, there is an urgent need to develop new generation adjuvants that will support the immunogenicity of antigens while not being toxic to the body. These adjuvants should be universal for many antigens and effective at reducing the doses of preparations and improving long-term stimulation of the systemic, cellular and mucosal immune response. It should also be noted that choosing the wrong adjuvant may reduce the effectiveness of a potential vaccine [97].

## 6. Is the Spike Protein of SARS-CoV-2 an Appropriate Antigen?

All IM vaccines that reached the market as well as all concepts of developing COVID-19 mucosal preparations rely on the native viral spike protein (S) of SARS-CoV-2 to induce potently neutralizing antibodies; the main antigen of inactivated virus vaccines is also the S protein, though in combination with other viral proteins found in the SARS-CoV-2 particle. The S protein is the primary target of antibodies capable of effectively neutralizing the virus. The S protein is also a hotspot of virus evolution, and its mutations reduce vaccine efficacy, causing waning immunity and necessitate revaccination. The problem of the need to update the vaccine against SARS-CoV-2 variants, as well as the apparent inevitability of future novel coronavirus outbreaks, indicate the necessity for a new-generation vaccine that induces broad and long-lasting immune protection [98]. Research on universal vaccines has been extensively conducted in the context of vaccines against influenza viruses [99]. In this approach, a strategy to combat virus adaptive capabilities is based on targeting conserved epitopes. A similar approach may be a strategy potentially effective against SARS-CoV-2 [98]. There are already some promising results concerning cross-reactive immune recognition induced by experimental vaccines. For example, Saunders et al. showed that intramuscular immunization of macaques with nanoparticles conjugated with the RBD of SARS-CoV-2 and adjuvanted with 3 M-052 and alum elicited cross-neutralizing antibody responses against SARS-CoV-2 (including the B.1.1.7, P.1 and B.1.351 variants), SARS-CoV and bat coronaviruses. These experiments are very promising in the context of universal coronavirus vaccine development.

## 7. COVID-19 Mucosal Vaccines Currently under Development

To date, a number of studies analyzing the validity and effectiveness of the use of mucosal vaccines in the fight against the COVID-19 pandemic have been carried out in animal models. One of the promising approaches was that of Du et al., who immunized mice with a preparation based on the recombinant receptor-binding domain from SARS-CoV-2 in combination with an adjuvant in the form of aluminum oxyhydroxide gel (known as Alhydrogel R). The induction of humoral, cellular and mucosal responses using three routes of vaccine administration was compared: intranasal, subcutaneous and intramuscular. The study showed that immunization by the intranasal route elicited effective humoral response and induced the strongest mucosal immunity. The nasal RBD vaccine induced an effective immune response on the surface of the nasal mucosa, lungs, intestines and genital tract. Considerable amounts of sIgA secreted by B cells from the nasal cavity and lung mucosa were also noted, which, according to the authors, may be the first line of defense against the virus infecting the respiratory tract, preventing it from invading cells [100].

In another study conducted in mice expressing the human angiotensin-converting enzyme 2 receptor, Hassan et al. used a preparation based on the chimpanzee adenovirus vector encoding the spike protein of SARS-CoV-2 (ChAd-SARS-CoV-2-S). A single intranasal dose of this vaccine induced a systemic and mucosal immune response, guaranteed high levels of neutralizing sIgA antibodies, and almost completely prevented SARS-CoV-2 infection in the upper and lower respiratory tract. However, when the same preparation was administered intramuscularly, no mucosal response was induced, and viral RNA was detected in the lungs. These results strongly suggest that intranasal administration of the vaccine creates a barrier against the virus, which blocks its replication and possibly its further transmission, and it is quite possible that the mice immunized in this way attained sterilizing immunity [101].

Additionally, the research results of Wu et al. suggest high efficacy of intranasal immunization against SARS-CoV-2 in animals. Mice vaccinated in this way with the replication-defective human type 5 adenovirus encoding the SARS-CoV-2 spike protein (Ad5-nCoV) were completely protected against upper and lower respiratory tract infection after the first dose of the formulation. One dose was also sufficient to build up immunity in the ferret upper respiratory tract. In both cases, the nasally administered preparation significantly reduced the level of virus replication in the upper respiratory tract [102].

An et al. used parainfluenza virus type 5 expressing the SARS-CoV-2 spike protein (termed CVXGA1) as a vaccine preparation. To demonstrate its effectiveness, they used two animal models: a model of severe disease in mice expressing angiotensin-converting enzyme 2 and a model of upper respiratory tract infection in ferrets. Contrary to the control, the presence of SARS-CoV-2 in lung and brain tissues was not detected in mice immunized with CVXGA1. Additionally, contrary to the control, mice immunized intranasally with this preparation showed only slight foci of cells with the N-protein present, indicating that these were the initial sites of infection, which did not subsequently develop. In addition, the vaccinated mice showed significantly less evidence of interstitial disease characteristic of viral pneumonia compared to the control without immunization. The results obtained by the authors also show that when confronted with a lethal dose of SARS-CoV-2, CVXGA1-immunized mice were 100% protected. Intranasal immunization of ferrets with the CVXGA1 vaccine generated high titers of anti-S IgG, anti-RBD IgG and neutralizing antibodies. Low levels of anti-S IgA in nasal washes were also detected. Following intranasal administration of SARS-CoV-2 to immunized ferrets, no viral RNA was detected in their nasal secretions, as was the case with the control sample. No viral genetic material was recorded in the trachea and lungs of vaccinated ferrets. An experiment investigating the possibility of blocking virus transmission in ferrets after immunization with CVXGA1 was performed by keeping healthy, unimmunized ferrets with CVXGA1-immunized ferrets infected with SARS-CoV-2. No virus was detected in healthy ferrets during the first 5 days after the start of such confrontation, suggesting that direct contact did not result in transmission of the virus. Seven days after the initiation of the challenge, healthy ferrets became infected; however, the authors theorized that this was due to open, adjacent cages and the presence of virus in the environment transmitted by unimmunized and infected ferrets present in the same room [103].

The COVAC-ND project by scientists at Utrecht University also focused on developing an effective intranasal vaccine against SARS-CoV-2. Reverse genetics technology was used to construct such a preparation, with Newcastle disease virus as a vector expressing the spike protein form of SARS-CoV-2, which is intended to elicit two types of immune responses, both mucosal and systemic. The results of preclinical studies of this preparation in an animal model have yet to be published [104]. Additionally, of note is the nasal aerosol vaccine based on proprietary outer membrane vesicle (OMV) click technology. This technology uses spherical particles (OMVs) released by gram-negative bacteria, which may contain multiple bacterial antigens that determine infection and survival inside the host. In the click platform, the induction of an immune response to a new antigen is supported by immunostimulatory peptides and proteins with which the surface of OMVs is decorated [105].

The approach presented in the work of Doremalen et al. also seems promising. It concerns the use of the already approved preparation ChAdOx 1nCoV (Oxford/AstraZeneca), administered intramuscularly, for mucosal (intranasal) immunization. Studies carried out on Syrian hamsters and rhesus macaques have shown that this vaccine is highly effective in inducing both mucosal and humoral immune responses and inhibiting virus transmission between individuals. In contrast to animals vaccinated intramuscularly with the same preparation, those immunized by the mucosal route showed significantly less viral load in swabs, and no viral RNA in the upper respiratory tract was observed. The data obtained from this work formed the basis for initiating a phase 1 clinical trial to investigate the safety and efficacy of such immunization in humans [106].

The studies mentioned above show that mucosal vaccines are a very promising approach in attempts to stop the COVID-19 pandemic and are definitely worthy of further analysis. This is reflected in the list of vaccine candidates published and updated by the WHO. Of the 137 vaccine preparations presented in it (20 December 2021), at various stages of clinical trials, 13 of them are administered orally or intranasally or in the form of an aerosol (Table 2) [2].

Researchers seek to develop mucosal vaccines based on various technologies that are already used in parenteral vaccines. Teams established in institutions, such as the University of Hong Kong, Vaxart Inc. or Bharat Biotech International Limited, have focused on using viral vectors as the basis of their preparations. Additionally, vaccines are based on inactivated or live attenuated virus, developed by Codagenix, Meissa Vaccines Inc. and Laboratorio Avi-Mex. The last group of preparations are protein subunit vaccines that target the S protein of the SARS-CoV-2 virus developed by the Center for Genetic Engineering and Biotechnology, Razi Vaccine and Serum Research Institute and Vaxform. Several of these vaccines (described later) are already being analyzed in the advanced 3rd phase of clinical trials. This is the penultimate step to confirm the effectiveness of a given drug, during which monitoring the tolerance and safety of the product is continued. This phase is thus referred to as the therapeutic confirmatory phase and involves thousands of patients with a specific disease. After successfully passing this phase, a given pharmaceutical can be approved for general use.

The DelNS1-2019-nCoV-RBD-OPT1 preparation developed by a team from the University of Hong Kong in collaboration with Xiamen University and Beijing Wantai Biological Pharmacy is an intranasal vaccine based on a replicating viral vector of influenza virus expressing the SARS-CoV-2 RBD. In contrast to simpler vaccine development pathways employing an attenuated SARS-CoV-2 virus, another genetically recombinant virus is used as a vector for the SARS-CoV-2 protein subunit (RBD) to elicit a broad protective immune response against COVID-19. It is not the only such approach in the context of the development of a vaccine preparation against SARS-CoV-2. The list of preclinical and clinical trials also includes an attenuated influenza vector vaccine (BiOCAD Global), a recombinant influenza A vaccine (Rospotrebnadzor) and an attenuated influenza virus vector expressing the S protein from SARS-CoV-2 (Fundação Oswaldo Cruz and Instituto Buntantan) [107].

Another candidate mucosal vaccine against SARS-CoV-2 that is very close to entering the pharmaceutical market is Razi CoV Pars, which was developed by the Razi Vaccine and Serum Institute of Iran. Ultimately, it is a recombinant spike protein vaccine administered in three doses, with the first two being intramuscular injections and the third being administered intranasally.

COVI-VAC, which is a mucosal vaccine formulation developed by Codagenix in collaboration with the Serum Institute of India, is also in an advanced phase of clinical trials. It is a codon-deoptimized live attenuated intranasal vaccine that induces a strong immune response against SARS-CoV-2 [108]. According to the information available on the authors’ website, COVI-VAC, a preparation based on the attenuated whole SARS-CoV-2 virus, is expected to induce immunity against a range of proteins associated with this virus, unlike vaccines targeting only the S protein of the virus and its subdomains. In preclinical tests of this preparation, one dose was sufficient to induce strong protection against SARS-CoV-2 in an animal model. The vaccine has also successfully passed tests to ensure its safety [109].

## 8. Discussion

Vaccines offer the strongest protection in the context of public health. However, to be successful, high rates of vaccination are required to establish herd immunity and stop the current COVID-19 pandemic. With a basic reproductive number equal to 5.7, the percentage of the population that must be vaccinated to achieve herd immunity is estimated to be as high as 82.5% [110]. Currently, a major limiting factor in reaching herd immunity across different populations is vaccine hesitancy [111]. In 2019, the WHO announced vaccine hesitancy as one of the top ten threats to global health. It was shown that the method of vaccine administration may strongly influence people’s opinion and hesitancy levels. When vaccination is administered by injection, it is plausible that the blood-injection-injury cluster of fears increases hesitancy. Research shows that in the UK adult population, blood-injection-injury fears may contribute to approximately 10% of cases of COVID-19 vaccine hesitancy [112]. Thus, addressing such fears may improve the effectiveness of vaccination campaigns. The solution to the problem may be the use of an effective mucosal vaccine, which may contribute not only to higher social acceptance resulting from the lack of need to administer them with a needle but also being much more effortless to dose, which would drastically reduce the cost of the work done by trained medical personnel and of vaccine storage. In addition to generating a systemic immune response, mucosal preparations are known to be effective in generating local immunity on the mucosa surface, which would effectively reduce transmission of the virus in the community. This indicates that it is worth intensifying research toward the development of a mucosal vaccine. Overall, vaccines based on new technology, such as virus DNA or RNA, as introduced in a relatively short time after the outbreak of the pandemic, were not accepted by a large part of society, which significantly hindered the rapid mass immunization needed to achieve herd immunity. Additionally, the fear of injection and the need for expensive cold-chain (up to −85 °C) logistics to deliver vaccines to distant parts of the globe are major problems to overcome. The development of an effective mucosal vaccine can help to overcome these problems. However, as we can conclude from the WHO data presented in this review, mucosal COVID-19 vaccines represent a minority (approximately 10%) of all formulations currently being analyzed in clinical trials. This is related to the difficulties encountered by researchers in developing an effective and safe vaccine administered directly to the mucosa. When constructing such preparations, a major problem seems to be making them nonhazardous to the organism while inducing an effective immune response to protect against COVID-19. For example, the use of an antigen in the form of a single protein (subunit vaccines) is associated with the need to support and increase levels of its immunogenicity in contact with the mucosa. In this case, high hopes are placed on the use of adjuvants, which are designed to significantly enhance the immune response to the antigen administered. Nevertheless, this is difficult to attain because there are only a few adjuvants approved as safe and effective in the context of their use in mucosal preparations. Another option may be the use of carriers, such as VLPs or ISCOMS, which have immunostimulatory properties themselves and greatly assist in enhancing an immune response to the carried antigen. Although the development of an effective vaccine is a major challenge, a number of promising vaccine preparations, which are presented in this review, are in the pipeline. Some of them are in the last phase of clinical trials preceding the registration application stage. Clinical studies of the mucosal administration of a preparation developed by the University of Oxford in cooperation with AstraZeneca, which is already used worldwide in immunization by the intramuscular route, only confirm the validity of the need to focus research on vaccines administered directly to the mucosa. The development of an effective preparation inducing a strong immune response at the point of virus entry into the organism could be a milestone in combating the COVID-19 pandemic.

## Figures and Tables

**Table 1:** Advantages and limitations of mucosal vaccines.

Mucosal Vaccines	
Advantages	Limitations
Easiness of administration	Lack of effective delivery systems
Less stringent preparations for purity requirements	Lack of safe and effective adjuvants to enhance the immunogenicity
Simple production and storage	Further development needed (optimal dose, clinical trials, new indications)
Problems related to needles are excluded	Poor induction of antigen-specific immune responses
Facilitated process of mass immunization	
Presumably induction of both systemic and local immune responses	
Eliminating cases of asymptomatic carriers of the pathogen	

**Table 2:** Landscape of candidate mucosal vaccines in clinical development based on WHO data [2].

Name of the Vaccine	Form	Developers	Route of Administration	Clinical Trials Phase
Covishield	Viral vector (non-replicating)	University of Oxford	IN	I
VXA-CoV2-1 Ad5	Viral vector (non-replicating)	Vaxart	ORAL	II
DelNS1-2019-nCoV-RBD-OPT 1	Viral vector (replicating)	University of Hong Kong; Xiamen University; Beijing Wantai Biological Pharmacy	IN	III
bacTRL-Spike	DNA based	Symvivo Corporation	ORAL	I
COVI-VAC	Live attenuated virus	Codagenix; Serum Institute of India	IN	III
CIGB-669	Protein subunit	Center for Genetic Engineering and Biotechnology	IN	I/II
Razi Cov Pars	Protein subunit	Razi Vaccine and Serum Research Institute	IM and IN	III
BBV154	Viral vector (non-replicating)	Bharat Biotech International Limited	IN	I
MV-014-2012	Live attenuated virus	Meissa Vaccines, Inc.	IN	I
-	Inactivated virus	Laboratorio Avi-Mex	IM or IN	I
CoV2-OGEN1	Protein subunit	USSF; Vaxform	ORAL	I
-	Viral vector (non-replicating)	CyanVac LLC	IN	I
-	Bacterial antigen-spore expression vector	DreamTec Research Limited	ORAL	NA
